# Adipocyte‐specific ET_B_ receptor overexpression induces obesity, insulin resistance, and dyslipidemia in high‐fat diet‐fed female mice

**DOI:** 10.14814/phy2.70561

**Published:** 2025-09-19

**Authors:** Bridget D. Konadu, Osvaldo Rivera‐Gonzales, Natalie A. Wilson, Madilyn Lewis, Hayley A. Murphy, Megumi F. Mills, Laura E. Coats, Joshua K. Michael, Jennifer R. Stapel, Joshua S. Speed

**Affiliations:** ^1^ Department of Physiology and Biophysics University of Mississippi Medical Center Jackson Mississippi USA

**Keywords:** adipose, endothelin, insulin resistance, obesity, sex differences

## Abstract

In rodent models of diet‐induced obesity, a metabolic syndrome‐like phenotype develops that is more pronounced in males compared to females. Our lab reported that endothelin‐1 (ET‐1) is increased in visceral adipose of high‐fat diet‐fed (HFD) mice where it inhibits adiponectin production through activation of the ET‐1 type B receptor (ET_B_). Further, adipocyte‐specific knockout of ET_B_ improves insulin sensitivity in HFD‐fed male mice. We report that males produce significantly more ET‐1 with higher expression of ET_B_ in visceral adipose compared to females. We hypothesized that adipocyte‐specific overexpression of the ET_B_ receptor (adET_B_OX) would abolish or attenuate protection against HFD observed in female mice. The data indicate that female adET_B_OX mice placed on HFD for 10 weeks had increased adiposity compared to floxed controls, while no detectable difference was observed between adET_B_OX and floxed controls fed NMD. Compared to NMD floxed control mice, insulin tolerance was impaired in adET_B_OX fed either NMD or HFD. Finally, HFD‐fed adET_B_OX had exacerbated dyslipidemia and insulin intolerance compared to floxed controls. These data indicate that reduced ET‐1 signaling on adipocytes at least partially mediates protection against HFD‐induced metabolic disease in female mice.

## 1 INTRODUCTION

The global rise in obesity is a significant health challenge with accompanying complications, such as insulin resistance, dyslipidemia, and chronic inflammation, all leading to increased morbidity and mortality rates worldwide (Fruh, 2017; Klop et al., 2013; Wen et al., 2022). These conditions arise from complex interactions between genetic, environmental, and hormonal factors, leading to excessive fat accumulation and impaired metabolic function (Flores‐Dorantes et al., 2020; Lin & Li, 2021). The diet‐induced obesity model in mice is a well‐established experimental approach for investigating the mechanisms underlying diet‐induced obesity and associated metabolic disturbances (Li et al., 2020; Stapleton et al., 2024; Wang & Liao, 2012). Notably, sex differences have been found in susceptibility to high‐fat diet (HFD)‐induced obesity and metabolic dysfunction, with female mice exhibiting a delayed onset of weight gain, insulin resistance, and dyslipidemia compared to males (Peng et al., 2020; Stranahan et al., 2023). Mechanisms contributing to the protection of females against HFD‐induced obesity are not fully understood.

One potential factor contributing to sex differences is the peptide endothelin‐1 (ET‐1) (Gohar et al., 2016; Gohar & Pollock, 2018; Kuczmarski et al., 2021). ET‐1 is a potent vasoconstrictor that has been implicated in the regulation of various physiological processes, including inflammation, cell proliferation, and hormone production. The effects of ET‐1 are mediated through two receptors: endothelin type A (ET_A_) and type B (ET_B_). Recent studies in our lab have shown that ET‐1 levels are elevated in the visceral adipose tissue of diet‐induced obese mice (Jenkins et al., 2020; Rivera‐Gonzalez et al., 2021). Furthermore, inhibiting ET‐1 receptors improves insulin and glucose tolerance and reduces cholesterol, triglycerides, and free fatty acids in male mice fed a high‐fat diet (HFD) for 10 weeks (Rivera‐Gonzalez et al., 2021). In addition, ET‐1 receptor antagonism reduces visceral adipose tissue inflammation, highlighting ET‐1's role in obesity‐related metabolic disturbances. More recently, our lab has shown that knockout of ET_B_ receptors in adipocytes attenuates obesity‐induced reductions in circulating adiponectin, improves insulin and glucose tolerance, and reduces cholesterol and triglycerides in male mice fed HFD (Rivera‐Gonzalez et al., 2024). These data suggest that ET_B_ receptors on adipocytes promote insulin resistance, which is partially through inhibition of adiponectin production in obese mice.

In the majority of the literature, including our recent published observations, only male mice were used because female mice do not exhibit impaired metabolic profiles and inflammation to the extent of male counterparts when fed HFD for 8–10 weeks (Ganz et al., 2014; Gupte et al., 2012; Singer et al., 2015); however, it was observed that female mice that overexpress ET_B_ receptors on adipocytes (adETBOX) gained weight similarly to wild type males (unpublished) on HFD. Therefore, we hypothesized that lower expression of ET‐1 and/or ET_B_ receptors in adipose tissue of female mice may contribute and play a crucial role in this protection by mitigating the adverse effects of ET‐1 on lipid metabolism and insulin signaling pathways. In the current study, we determined if female mice overexpressing the human ET_B_ on adipocytes (adET_B_OX) have impaired insulin resistance and dyslipidemia after 10 weeks of HFD feeding, providing valuable insight into the role of ET‐1 and ET_B_ receptors in adipose tissue and their contribution to the differential susceptibility of female mice to diet‐induced metabolic dysfunctions.

## 2 MATERIALS AND METHODS

### 2.1 Animal husbandry and generation of models

Mice were bred on the same rack and room at the University of Mississippi Medical Center (UMMC) under 12‐h light/12‐h dark conditions and allowed food and water ad libitum. Female mice overexpressing the human ET_B_ on adipocytes (adET_B_OX) were generated as previously described (Rivera‐Gonzalez et al., 2024). Cre recombination in adET_B_OX was visualized by mCherry expression only found in adipose tissue. Wild type (WT) mice used in each experiment were Cre^−^ littermates with intact floxed sites from the respective colony and will be referred to as floxed or Control (Con). At 8 weeks of age, mice were randomized and individually housed into two groups (female WT mice or adET_B_OX); female WT mice or adET_B_OX were fed on normal diet‐fed (NMD; 12.6% kcal fat, 30% kcal carbohydrate, Envigo, TD.05230) (*n* = 7) or high‐fat diet‐fed (HFD; 45% kcal fat, 42% kcal carbohydrate, Envigo TD.88137) for 8 weeks. Diet was continued as glucose tolerance, insulin tolerance, and euthanasia were performed with 3 days between each. Mice were euthanized under isoflurane anesthesia after a 6‐h fast in clean cages beginning at zeitgeber time 1–2, and tissues were collected. All assays were conducted on the same mice and on the same day. All protocols were approved by the Institutional Animal Care and Use Committee at UMMC.

### 2.2 Body composition analysis

Lean mass, fat mass, and total body water composition were measured using Echo MRI (4‐in‐1 EchoMRI‐900™, Echo Medical System, Houston, TX) at weeks 0, 4, and 8 following the start of the diet.

### 2.3 Insulin and glucose tolerance

Mice were fasted for 6 h beginning at ZT 1–2 prior to the intraperitoneal insulin tolerance test (ITT) and oral glucose tolerance test (OGTT). Glucose was measured using a glucometer (Accu‐Chek® Guide) via tail vein. For ITT, insulin (0.75 IU/kg of lean mass) was injected into the peritoneal cavity. For OGTT, dextrose (2 g/kg lean mass) was administered via oral gavage through a 22‐gauge gavage. Two glucose measurements were acquired at baseline or time 0, 15, 30, 60, 90, and 120 min following insulin injection or glucose bolus. Tests were performed 3 days apart and similar to guidelines set forth by Benede‐Ubieto et al. (2020). Since only a small drop of blood was taken at each time point, 3 days rather than a week was allowed for recovery. The area under the curve was calculated using GraphPad Prism statistical software where baseline was set at time = 0 for each individual mouse.

### 2.4 Biochemical analysis

Blood was collected under anesthesia via cardiac puncture using a 22 g needle with a 1 mL syringe coated with EDTA (0.5 M) and immediately placed on ice. Blood was spun at 1000 RCF for 10 min at 4°C to separate plasma. Blood chemistry, including cholesterol (CHOL), high‐density lipoprotein (HDL), low‐density lipoprotein (LDL), nonesterified fatty acids (NEFA), and triglycerides (TRIG) was analyzed via the Vet Axcel® chemistry analyzer (Alfa Wasserman). Plasma insulin and adiponectin concentrations were measured by mouse enzyme‐linked immunoassay (ELISA) (Crystal Chem, 90080, 80569) according to the manufacturer's protocols.

### 2.5 Droplet digital PCR (ddPCR)

Total RNA was isolated using the Direct‐zol™ RNA miniprep kit (Zymo, R2063‐A) following the manufacturer's protocol. RNA was reversed transcribed with iScript Reverse Transcription Supermix (Bio‐Rad) to generate cDNA. Droplets were generated using the Bio‐Rad Auto DG. PCR was carried out with ddPCR Supermix for probes with Applied Biosystems Taqman primer assays (Primers with their Taqman assay ID included endothelin B receptor or *Ednra*, *Mm01243722_m1*, endothelin B receptor or *Ednrb*, *Mm00432989_m1*, and ET‐1 or *Edn1*, *Mm00438659_m1*). Droplets were counted by the Bio‐Rad QX200. Gene expression was quantified with the Bio‐Rad QuantaSoft software, and results were expressed as copies /25 ng RNA.

### 2.6 Randomization and statistics

All data are expressed as mean ± SEM. Data were tested for statistical significance by one‐way ANOVA for one variable datasets or two‐way repeated measure ANOVA (ITT and OGTT). Tukey's post hoc test was used to compare groups. *p* < 0.05 was considered statistically significant. All graphs and statistical analyses were performed using GraphPad Prism (Version 10.3.1).

## 3 RESULTS

### 3.1 Female mice have significantly reduced ET_B_ receptor mRNA expression in visceral adipose compared to males

**FIGURE 1:**
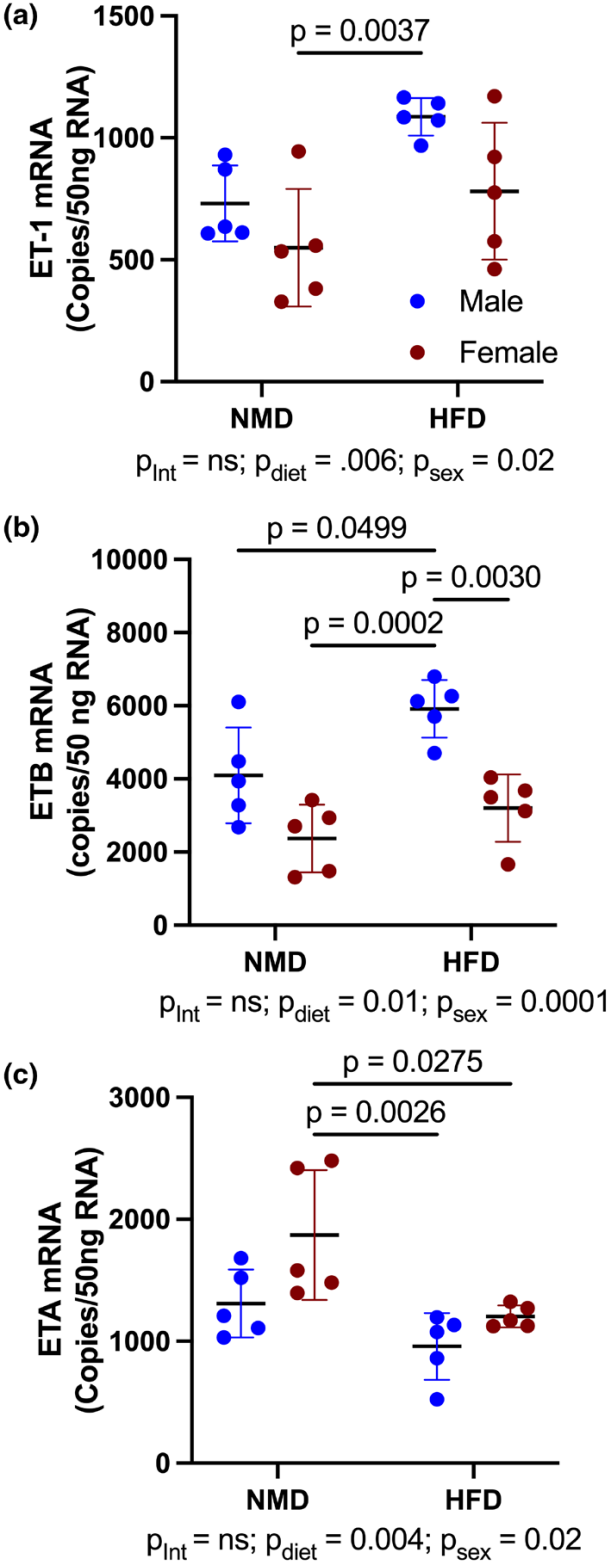
Female mice have significantly reduced adipocyte expression of ET_B_ receptor. (a) ET‐1, (b) ET_B_ receptor, and (c) ET_A_ receptor mRNA expression in visceral (gonadal) adipose tissue of male and female C57Bl6 mice fed NMD or HFD for 10 weeks. Data were analyzed using a two‐way ANOVA with Tukey's post hoc test for individual groups. Data are expressed as mean ± SD. *p* Values represent results from multiple comparisons post hoc analysis.

To establish whether sex differences in ET‐1, ET_A_, and ET_B_ receptor expression exist in adipose tissue, we assessed transcripts by ddPCR in visceral gonadal adipose of male and female mice. Our findings revealed significantly lower ET‐1 expression in visceral adipose of female adET_B_OX mice (*p*_Int_ = ns; *p*_diet_ = 0.006; *p*_sex_ = 0.02). In males, ET_B_ expression was significantly increased in male mice under HFD compared to NMD (*p* = 0.049), whereas no diet‐related differences were observed in females. Notably, ET_B_ expression was significantly lower in females compared to males under HFD conditions (*p* = 0.003, Figure 1b). There was no significant difference in ET_A_ expression between NMD male and female mice; however, HFD significantly reduced ET_A_ expression in female mice (*p* = 0.0275, Figure 1c).

### 3.2 Overexpression of ET_B_ receptors on adipocytes increases body weight and adiposity in HFD‐fed female mice

**FIGURE 2:**
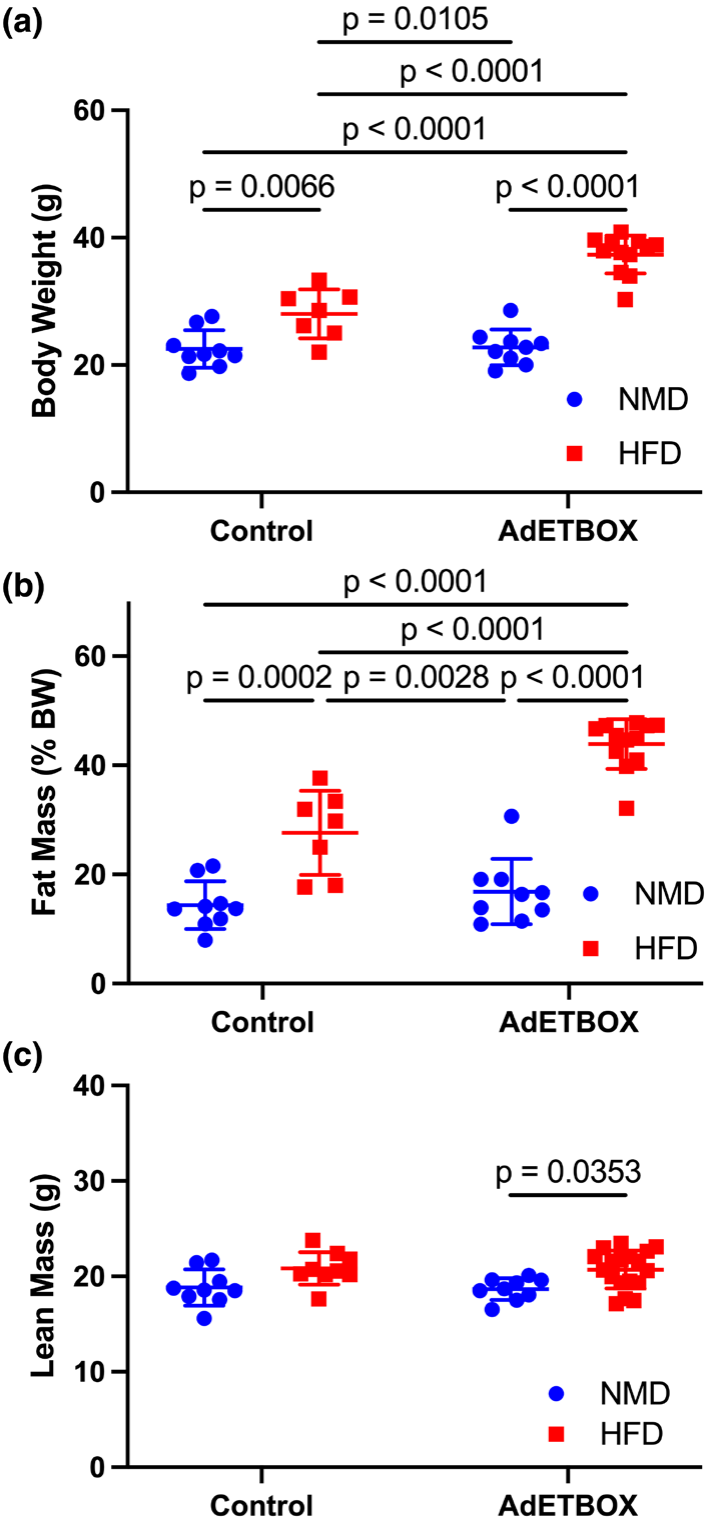
Body weight and fat mass are significantly increased by overexpression of ET_B_ receptor by adipocytes in female mice. (a) Body weight (grams), (b) fat mass (percent of body weight), and (c) lean mass (percent of body weight) in female floxed control or adET_B_OX mice fed NMD or HFD after 8 weeks of NMD or HFD. Data were analyzed by two‐way ANOVA with post hoc Tukey's post hoc test to compare each group. Data are expressed as mean ± SD. *p* Values represent results from multiple comparisons post hoc analysis. ANOVA tables: 2a, *p*_Interaction_ < 0.001, *p*_gen_ < 0.001, *p*_diet_ < 0.001; 2b, *p*_Interaction_ = 0.0008, *p*_gen_ < 0.0001, *p*_diet_ < 0.0001; 2c, *p*_Interaction_ = 0.97, *p*_gen_ = 0.79, *p*_diet_ = 0.0007.

To examine the impact of ET_B_ receptor overexpression on body composition in female adET_B_OX mice, echo MRI was used to measure fat and lean mass. There was no significant difference in body weight between control and adETBOX mice fed NMD; however, HFD‐fed adET_B_OX mice had significantly higher body weight (28.03 ± 3.86 g vs. 37.43 ± 1.52 g; control vs. adET_B_OX; Figure 2a, *p* < 0.0001) and fat mass (27.6 ± 7.7 %BW vs. 43.9 ± 4.6% of BW; control vs. adET_B_OX; Figure 2b, *p* < 0.0001) compared to control HFD‐fed mice. In addition, HFD‐fed mice had a significant increase in percent lean mass of body weight, but there was no effect of genotype (Figure 2c, *p*_Interaction_ = 0.97, *p*_gen_ = 0.79, and *p*_diet_ = 0.0007).

### 3.3 Adipocyte ET_B_ receptor overexpression abolishes “protection” from HFD‐induced insulin resistance in female mice

**FIGURE 3:**
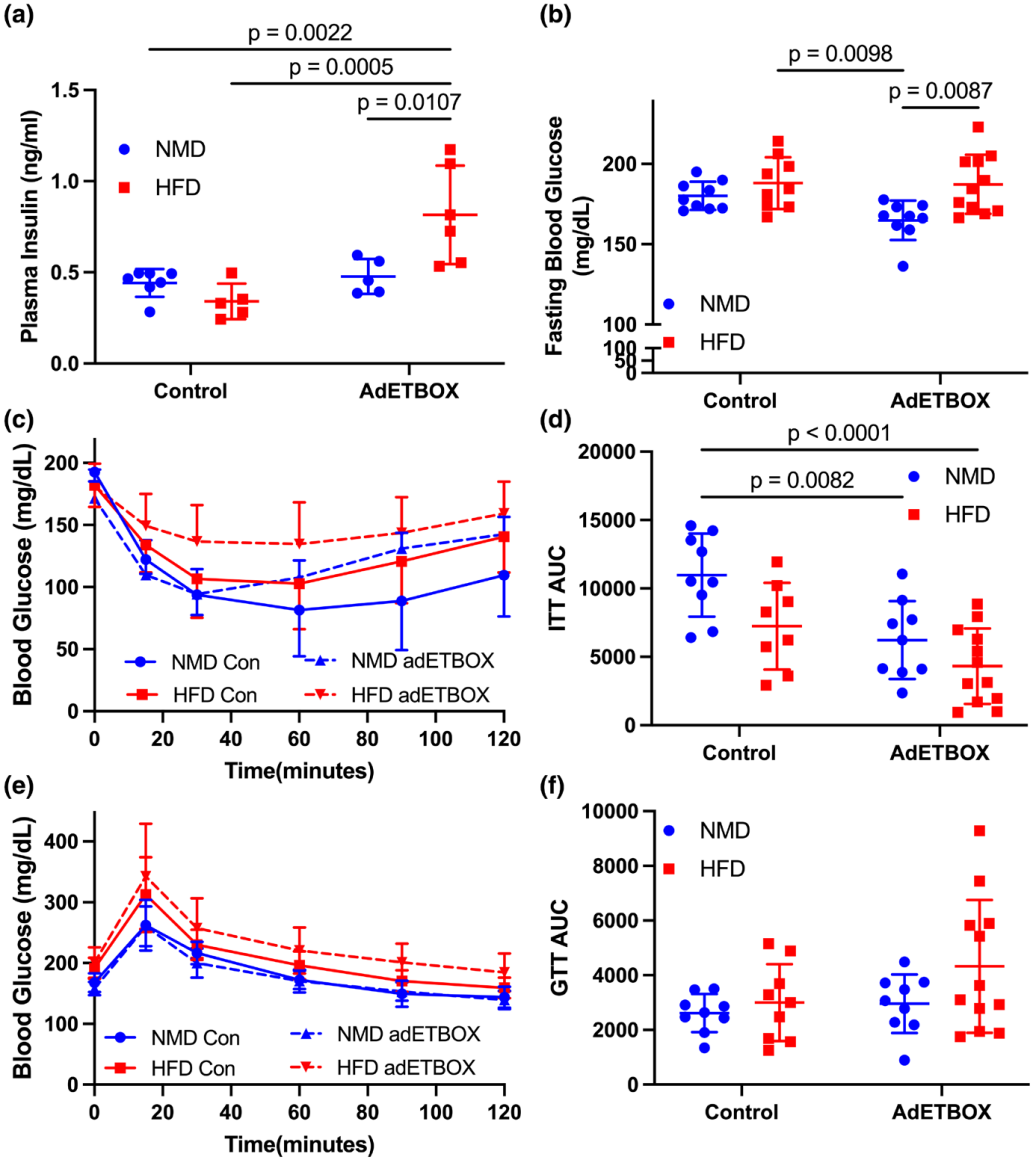
Female adET_B_OX receptor overexpression mice. (a) Plasma insulin concentration and (b) fasting blood glucose from female floxed control or adET_B_OX mice fed NMD or HFD for 10 weeks. (c) Insulin tolerance test (ITT) and (d) Area under the curve (AUC) followed by (e) oral glucose tolerance test (OGTT) and (f) OGTT AUC. ITT and GTT were performed between 8 and 10 weeks of NMD or HFD feeding per methods. Insulin, fasting glucose, and AUC were analyzed using a two‐way ANOVA with post hoc Tukey's test between individual groups. Data are expressed as mean ± SD. *p* Values represent results from multiple comparisons post hoc analysis. ANOVA results: ITT *p*_time_ < 0.0001, *p*_gen_ = 0.03, *p*_diet_ < 0.001, *p*_time × gen × diet_. GTT *p*_time_ < 0.0001, *p*_gen_ = 0.22, *p*_diet_ <0.0001, *p*_time × gen × diet_ = 0.94.

To investigate the impact of ET_B_ receptor overexpression in adipocytes on glucose metabolism in female mice, we measured fasting insulin and glucose and performed ITT and GTT. There was no detectable difference in fasting insulin in control NMD or HFD‐fed mice or NMD fed adET_B_OX mice (Figure 3a). Interestingly, fasting insulin was significantly elevated in HFD‐fed adET_B_OX mice (Figure 3a). As expected, HFD significantly increased fasting glucose, which was calculated by averaging the baseline glucose from ITT and GTT, although there was no detectable effect of genotype (Figure 3b). Post hoc analysis showed that fasting blood glucose was significantly lower in NMD fed adET_B_OX compared to both floxed control and adET_B_OX fed HFD (Figure 3b). Furthermore, adET_B_OX mice had significantly impaired insulin tolerance whether on NMD or HFD (ANOVA: *p*_time_ < 0.0001, *p*_gen_ = 0.03, *p*_diet_ < 0.001, *p*_time × gen × diet_. GTT *p*_time_ < 0.0001, *p*_gen_ = 0.22, *p*_diet_ < 0.0001, *p*_time × gen × diet_ = 0.94). HFD exacerbated impaired ITT (Figure 3d, AUC 10978 ± 3034 vs. 6229 ± 2847 vs. 7245 ± 3167 vs. 4320 ± 2762, NMD control vs. NMD adETBOX vs. control HFD vs. HFD ET_B_OX). No significant differences were found as an effect of genotype or diet. Glucose tolerance was impaired by HFD feeding in both genotypes; (Figure 3e,f, AUC *p*_int_ = 0.36, *p*_gen_ = 0.12, *p*_diet_ = 0.11).

### 3.4 ET_B_ receptor overexpression on adipocytes worsens dyslipidemia in response to HFD in female mice

**FIGURE 4:**
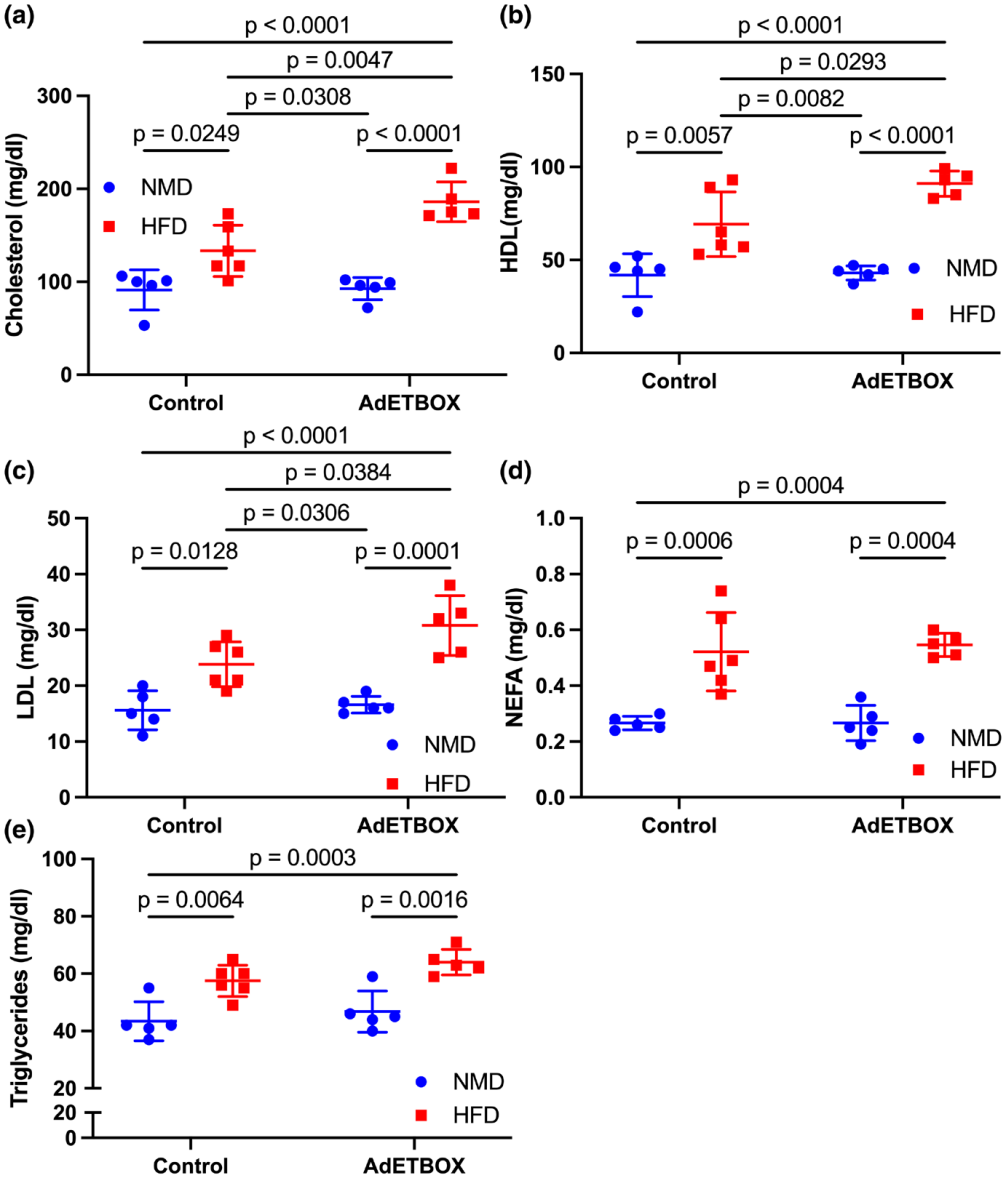
Adipocyte ET_B_ overexpression in female mice increases dyslipidemia following feeding. Plasma concentration of (a) cholesterol, (b) high‐density lipoprotein (HDL), (c) low‐density lipoprotein (LDL), (d) nonesterified free fatty acids (NEFA), and (e) triglycerides in female floxed control or adET_B_OX mice fed NMD or HFD for 10 weeks. Statistical analyses were performed by two‐way ANOVA with Tukey's post hoc test between individual groups. Data are expressed as mean ± SD. *p* Values represent results from multiple comparisons post hoc analysis. ANOVA tables: 4a, *p*_Interaction_ = 0.016, *p*_gen_ < 0.012, *p*_diet_ < 0.0001; 4b, *p*_Interaction_ = 0.058, *p*_gen_ < 0.037, *p*_diet_ < 0.0001; 4c, *p*_Interaction_ = 0.096, *p*_gen_ < 0.03, *p*_diet_ < 0.0001; 4d, *p*_Interaction_ = 0.57, *p*_gen_ < 0.079, *p*_diet_ < 0.0001; 4e, *p*_Interaction_ = 0.75, *p*_gen_ < 0.075, *p*_diet_ < 0.0001.

We subsequently investigated whether specific ET_B_ receptor overexpression impacted circulating lipids. Our findings indicate that HFD significantly raised circulating cholesterol levels in both floxed control and adETBOX mice, an effect that was significantly exacerbated in adETBOX (Figure 4a, *p*_int_ = 0.02, *p*_gen_ = 0.01, *p*_diet_ < 0.0001). The differences in cholesterol were partially due to increases in both HDL‐ and LDL‐cholesterol (Figure 4b,c, HDL *p*_int_ = 0.06, *p*_gen_ = 0.04, *p*_diet_ < 0.0001; LDL *p*_int_ = 0.10, *p*_gen_ = 0.03, *p*_diet_ < 0.0001). HFD increased both non‐NEFA and triglycerides; however, no significant effect was observed by genotype (Figure 4e,f, NEFA *p*_int_ = 0.75, *p*_gen_ = 0.75, *p*_diet_ < 0.0001; triglycerides *p*_int_ = 0.57, *p*_gen_ = 0.08, *p*_diet_ <0.0001).

## 4 DISCUSSION

Elevated levels of endothelin‐1 (ET‐1) are associated with obesity and insulin resistance in humans and increased in visceral adipose of obese mice fed a high‐fat diet (HFD) (Ferri et al., 1995; Ottosson‐Seeberger et al., 1997; Rivera‐Gonzalez et al., 2021). In this study, we examined differences in ET‐1 and ET‐1 receptor expression in visceral adipose tissue of male and female mice. In addition, we examined the effects of adipocyte‐specific ET_B_ overexpression on HFD‐induced changes in lipid and glucose homeostasis in female mice. Our findings demonstrated a significant sex‐specific difference in ET‐1 and ET_B_ receptor expression in visceral adipose, with female mice exhibiting significantly lower levels of both compared to males. Importantly, the metabolic “protection” exhibited by females compared to males against 8‐week HFD‐induced insulin resistance and dyslipidemia was abolished in female adET_B_OX mice. The overexpression resulted in a significant impairment in insulin tolerance and increased plasma cholesterol following HFD, highlighting a critical role of ET_B_ receptor in adipocytes to promote metabolic dysfunction in HFD‐induced obesity.

Our lab recently demonstrated that genetic deletion of ET_B_ specifically in adipocytes of male mice significantly attenuated glucose and insulin resistance with no significant difference in whole body fat content compared to controls, under a similar HFD feeding protocol as the current study. In contrast, our findings indicate that overexpression of ET_B_ receptors in female mice increases adiposity and exacerbates impaired ITT and hyperinsulinemia in mice fed HFD. Together, the data indicate that ET_B_ receptor activation in adipocytes plays a key role in promoting obesity‐related adipocyte dysfunction and subsequent development of metabolic syndrome. These results are consistent with previous studies showing that global knockout of the ET_B_ receptor in male mice and rats improves insulin resistance, dyslipidemia, and increased accumulation of white adipose tissue (WAT) (Feger et al., 2024; Rivera‐Gonzalez et al., 2024). This evidence collectively highlights the potential importance of ET_B_ signaling pathways in regulating adipocyte function and systemic metabolic health in the setting of obesity.

It is well known that ET‐1 plays an important role in promoting insulin resistance through ET_A_ receptor (Sarafidis & Bakris, 2007), and our current data support previous reports of an important ET_B_ receptor‐mediated pathway in adipocytes (Feger et al., 2024; Rivera‐Gonzalez et al., 2024). Our findings also indicate that overexpression of the adipocyte ET_B_ receptor in female mice significantly impaired insulin tolerance under NMD conditions (Figure 3e,f) and this impairment was further exacerbated by HFD. Further, no significant difference was observed in the oral glucose tolerance test, suggesting that glucose disposal capacity was preserved despite impaired insulin tolerance and hyperinsulinemia. Our results align with previous studies showing that male adipocyte ET_B_ receptor‐deficient mice are protected from insulin resistance after HFD feeding (Feger et al., 2024; Polak et al., 2018). In the same study, it was reported that HFD feeding elevates basal circulating insulin levels of control male mice, but not control female mice. We observed that females overexpressing the ET_B_ receptor exhibited increased fasting insulin and impaired insulin tolerance following 10 weeks of HFD feeding. This insulin‐resistant phenotype correlated with increased adiposity, suggesting more work is needed to determine if loss of protection from HFD feeding in control female mice is due to increased adiposity or changes in other circulating factors that may impact whole body insulin sensitivity. Nevertheless, these data suggest that ET_B_ receptor overexpression in adipocytes contributes to insulin resistance in HFD‐induced obesity, highlighting a potential mechanism by which ET_B_ receptor signaling influences metabolic health under obesogenic conditions.

A major finding of the current study is that female adET_B_OX mice have significantly more adiposity after high‐fat feeding compared to controls. In fact, control female mice fed on HFD exhibit a modest 25 percent more body weight compared to control females on NMD; however, female adET_B_OX mice had a significant 64% more body weight, attributable to more fat mass. A similar phenotype was observed in male mice shown in two previous reports by our group (Rivera‐Gonzalez et al., 2021, 2024). Importantly, no difference was observed between female control or adET_B_OX fed normal diet. These data suggest that ET_B_ receptor overexpression may override the protective effect observed in females, which we report here have lower visceral adipose ET‐1 and ET_B_ receptor expression compared to males, even in the context of HFD. This shift in body composition corresponds with a notable rise in circulating lipids, including cholesterol, HDL, and LDL in the adET_B_OX female mice. Interestingly, in women, responsiveness to ET‐1 vasoconstriction has been associated with high LDL, in contrast to men, as highlighted by prior studies (Romerio et al., 2000). It has been demonstrated that ET_B_ receptor protein and mRNA expression can be induced by LDL in primary cultures of human umbilical vein endothelial cells (HUVECs) (Muller et al., 2006), which may explain the differences in LDL levels observed between the control and overexpression groups and supports the link between ET_B_ receptor signaling and dysregulated lipid metabolism. More studies are required to determine the mechanisms by which increased ET_B_ signaling increases adiposity in female HFD‐fed mice.

Previous literature has demonstrated that ET‐1 receptor function differs between males and females, especially in controlling vascular and kidney function (Kittikulsuth et al., 2013). The activation of vascular ET_B_ receptors is known to cause vasorelaxation; however, ET‐1/ET_B_ regulation of vascular tone may differ between men and women. For example, in men, ET_B_ receptors on vascular smooth muscle cells may contribute more to vasoconstriction, thereby offsetting the vasodilatory properties of ET_B_ receptors on endothelial cells (Kellogg Jr. et al., 2001). This sex‐dependent function suggests that ET_B_ receptors may play distinct roles in disease processes between men and women. In this study, we observed significantly lower ET‐1 and ET_B_ receptor expression in visceral adipose tissue of female mice compared to male mice, especially under high‐fat diet‐induced obesity conditions. In fact, ET‐1 and ET_B_ expression were significantly increased in visceral adipose of HFD‐fed male mice. In contrast, there was no significant difference in ET‐1 and ET_B_ expression between NMD and HFD‐fed female mice. These data may suggest an important role for ET‐1 in promoting insulin resistance in male, but not female mice fed HFD, at least in the early stages of HFD‐induced obesity. Supporting this theory, our lab reported that adipocyte‐specific knockout of ET_B_ receptors using the same adiponectin Cre used to generate adET_B_OX mice significantly improved insulin and glucose tolerance in HFD‐fed male mice (Rivera‐Gonzalez et al., 2024). A possible explanation for the loss of “protection” against HFD‐induced metabolic dysfunction in female adETBOX mice is the influence of estradiol (E2). ET_B_ receptor activation has been linked to greater vasodilation in young women in the presence of E2 (Shoemaker et al., 2021), thereby contributing to microvascular endothelial function. In addition, estradiol has been shown to inhibit ET‐1 synthesis by endothelial cells and in humans administered 17β‐estradiol (Dubey et al., 2001; Rosano et al., 2007). Further investigation is needed to clarify whether sex hormones alter ET_B_ receptor function in adipocytes.

## 5 CONCLUSIONS AND PERSPECTIVES

The majority of the literature aimed at determining mechanisms of metabolic dysfunction in obesity was performed in male mice because males are more susceptible to the development of metabolic syndrome like pathophysiology, especially in HFD models of obesity (Pettersson et al., 2012). To our knowledge, sex differences in ET‐1 and ET_B_ signaling in the setting of obesity have not been reported. Our current data suggest that protection against obesity‐induced phenotypes is at least partially mediated by lower ET‐1/ET_B_ signaling in adipose in female mice. These data also support the novel findings that ET‐1 in adipose tissue impairs adipocyte function and consideration of metabolic phenotypes may be warranted in the design of future clinical trials of ET‐1 receptor antagonists.

## AUTHOR CONTRIBUTIONS

BDK, ORG, and JSS conceived and designed research; BDK, ORG, NAW, ML, MFM, HAM, JKM, JRS, LEC, and JSS performed experiments. BDK, ORG, and JSS analyzed data, interpreted results of experiments, prepared figures, and drafted the manuscript. BDK, ORG, NAW, ML, MFM, HAM, JKM, JRS, LEC, and JSS edited and revised manuscript and approved the final version of the manuscript.

## FUNDING INFORMATION

This work was supported by grants from the National Institute of General Medical Sciences (U54 GM115428) to JSS, the National Institute of General Medical Sciences (P30 GM149404) to the UMMC Department of Physiology, the National Heart, Lung, and Blood Institute (T32 HL105324) to JSS, and the National Institute on Diabetes, Digestive, and Kidney Diseases (R01 DK124327) to JSS.

## CONFLICT OF INTEREST STATEMENT

The authors have no conflicts of interest.

## ETHICS STATEMENT

All animal protocols were approved the the Institutional Animal Care and Use committee at the University of Mississippi Medical Center. Data will provide upon request to the corresponding author.

## Data Availability

Data will be made available upon request.

## References

[phy270561-bib-0001] Benede‐Ubieto, R. , Estevez‐Vazquez, O. , Ramadori, P. , Cubero, F. J. , & Nevzorova, Y. A. (2020). Guidelines and considerations for metabolic tolerance tests in mice. Diabetes, Metabolic Syndrome and Obesity: Targets and Therapy, 13, 439–450. 10.2147/DMSO.S234665 32110077 PMC7038777

[phy270561-bib-0002] Dubey, R. K. , Jackson, E. K. , Keller, P. J. , Imthurn, B. , & Rosselli, M. (2001). Estradiol metabolites inhibit endothelin synthesis by an estrogen receptor‐independent mechanism. Hypertension, 37(2 Pt 2), 640–644. 10.1161/01.hyp.37.2.640 11230349

[phy270561-bib-0003] Feger, M. , Meier, L. , Strotmann, J. , Hoene, M. , Vogt, J. , Wisser, A. , Hirschle, S. , Kheim, M. J. , Hocher, B. , Weigert, C. , & Foller, M. (2024). Endothelin receptor B‐deficient mice are protected from high‐fat diet‐induced metabolic syndrome. Molecular Metabolism, 80, 101868. 10.1016/j.molmet.2023.101868 38159882 PMC10825011

[phy270561-bib-0004] Ferri, C. , Bellini, C. , Desideri, G. , Di Francesco, L. , Baldoncini, R. , Santucci, A. , & De Mattia, G. (1995). Plasma endothelin‐1 levels in obese hypertensive and normotensive men. Diabetes, 44(4), 431–436. 10.2337/diab.44.4.431 7698512

[phy270561-bib-0005] Flores‐Dorantes, M. T. , Diaz‐Lopez, Y. E. , & Gutierrez‐Aguilar, R. (2020). Environment and Gene Association with obesity and their impact on neurodegenerative and neurodevelopmental diseases. Frontiers in Neuroscience, 14, 863. 10.3389/fnins.2020.00863 32982666 PMC7483585

[phy270561-bib-0006] Fruh, S. M. (2017). Obesity: Risk factors, complications, and strategies for sustainable long‐term weight management. Journal of the American Association of Nurse Practitioners, 29(S1), S3–S14. 10.1002/2327-6924.12510 29024553 PMC6088226

[phy270561-bib-0007] Ganz, M. , Csak, T. , & Szabo, G. (2014). High fat diet feeding results in gender specific steatohepatitis and inflammasome activation. World Journal of Gastroenterology, 20(26), 8525–8534. 10.3748/wjg.v20.i26.8525 25024607 PMC4093702

[phy270561-bib-0008] Gohar, E. Y. , Giachini, F. R. , Pollock, D. M. , & Tostes, R. C. (2016). Role of the endothelin system in sexual dimorphism in cardiovascular and renal diseases. Life Sciences, 159, 20–29. 10.1016/j.lfs.2016.02.093 26939577 PMC4992599

[phy270561-bib-0009] Gohar, E. Y. , & Pollock, D. M. (2018). Sex‐specific contributions of endothelin to hypertension. Current Hypertension Reports, 20(7), 58. 10.1007/s11906-018-0856-0 29884912 PMC6921242

[phy270561-bib-0010] Gupte, M. , Thatcher, S. E. , Boustany‐Kari, C. M. , Shoemaker, R. , Yiannikouris, F. , Zhang, X. , Karounos, M. , & Cassis, L. A. (2012). Angiotensin converting enzyme 2 contributes to sex differences in the development of obesity hypertension in C57BL/6 mice. Arteriosclerosis, Thrombosis, and Vascular Biology, 32(6), 1392–1399. 10.1161/ATVBAHA.112.248559 22460555 PMC3355213

[phy270561-bib-0011] Jenkins, H. N. , Rivera‐Gonzalez, O. , Gibert, Y. , & Speed, J. S. (2020). Endothelin‐1 in the pathophysiology of obesity and insulin resistance. Obesity Reviews, 21(12), e13086. 10.1111/obr.13086 32627269 PMC7669671

[phy270561-bib-0012] Kellogg, D. L., Jr. , Liu, Y. , & Pergola, P. E. (2001). Selected contribution: Gender differences in the endothelin‐B receptor contribution to basal cutaneous vascular tone in humans. Journal of Applied Physiology (1985), 91(5), 2407–2411. 10.1152/jappl.2001.91.5.2407 11641388

[phy270561-bib-0013] Kittikulsuth, W. , Looney, S. W. , & Pollock, D. M. (2013). Endothelin ET(B) receptors contribute to sex differences in blood pressure elevation in angiotensin II hypertensive rats on a high‐salt diet. Clinical and Experimental Pharmacology and Physiology, 40(6), 362–370. 10.1111/1440-1681.12084 23713708 PMC3693481

[phy270561-bib-0014] Klop, B. , Elte, J. W. , & Cabezas, M. C. (2013). Dyslipidemia in obesity: Mechanisms and potential targets. Nutrients, 5(4), 1218–1240. 10.3390/nu5041218 23584084 PMC3705344

[phy270561-bib-0015] Kuczmarski, A. V. , Welti, L. M. , Moreau, K. L. , & Wenner, M. M. (2021). ET‐1 as a sex‐specific mechanism impacting age‐related changes in vascular function. Frontiers in Aging, 2, 727416. 10.3389/fragi.2021.727416 35822003 PMC9261354

[phy270561-bib-0016] Li, J. , Wu, H. , Liu, Y. , & Yang, L. (2020). High fat diet induced obesity model using four strains of mice: Kunming, C57BL/6, BALB/c and ICR. Experimental Animals, 69(3), 326–335. 10.1538/expanim.19-0148 32188837 PMC7445062

[phy270561-bib-0017] Lin, X. , & Li, H. (2021). Obesity: Epidemiology, pathophysiology, and therapeutics. Frontiers in Endocrinology, 12, 706978. 10.3389/fendo.2021.706978 34552557 PMC8450866

[phy270561-bib-0018] Muller, G. , Catar, R. A. , Niemann, B. , Barton, M. , Knels, L. , Wendel, M. , & Morawietz, H. (2006). Upregulation of endothelin receptor B in human endothelial cells by low‐density lipoproteins. Experimental Biology and Medicine, 231(6), 766–771. 16740996

[phy270561-bib-0019] Ottosson‐Seeberger, A. , Lundberg, J. M. , Alvestrand, A. , & Ahlborg, G. (1997). Exogenous endothelin‐1 causes peripheral insulin resistance in healthy humans. Acta Physiologica Scandinavica, 161(2), 211–220. 10.1046/j.1365-201X.1997.00212.x 9366964

[phy270561-bib-0020] Peng, C. , Xu, X. , Li, Y. , Li, X. , Yang, X. , Chen, H. , Zhu, Y. , Lu, N. , & He, C. (2020). Sex‐specific association between the gut microbiome and high‐fat diet‐induced metabolic disorders in mice. Biology of Sex Differences, 11(1), 5. 10.1186/s13293-020-0281-3 31959230 PMC6971877

[phy270561-bib-0021] Pettersson, U. S. , Walden, T. B. , Carlsson, P. O. , Jansson, L. , & Phillipson, M. (2012). Female mice are protected against high‐fat diet induced metabolic syndrome and increase the regulatory T cell population in adipose tissue. PLoS One, 7(9), e46057. 10.1371/journal.pone.0046057 23049932 PMC3458106

[phy270561-bib-0022] Polak, J. , Punjabi, N. M. , & Shimoda, L. A. (2018). Blockade of endothelin‐1 receptor type B ameliorates glucose intolerance and insulin resistance in a mouse model of obstructive sleep apnea. Frontiers in Endocrinology, 9, 280. 10.3389/fendo.2018.00280 29896159 PMC5986958

[phy270561-bib-0023] Rivera‐Gonzalez, O. , Mills, M. F. , Konadu, B. D. , Wilson, N. A. , Murphy, H. A. , Newberry, M. K. , Hyndman, K. A. , Garrett, M. R. , Webb, D. J. , & Speed, J. S. (2024). Adipocyte endothelin B receptor activation inhibits adiponectin production and causes insulin resistance in obese mice. Acta Physiologica, 240(10), e14214. 10.1111/apha.14214 39096077 PMC11421981

[phy270561-bib-0024] Rivera‐Gonzalez, O. , Wilson, N. A. , Coats, L. E. , Taylor, E. B. , & Speed, J. S. (2021). Endothelin receptor antagonism improves glucose handling, dyslipidemia, and adipose tissue inflammation in obese mice. Clinical Science, 135(14), 1773–1789. 10.1042/CS20210549 34278410 PMC8650556

[phy270561-bib-0025] Romerio, S. C. , Linder, L. , Flammer, J. , & Haefeli, W. E. (2000). Correlation between apolipoprotein B and endothelin‐1‐induced vasoconstriction in humans. Peptides, 21(6), 871–874. 10.1016/s0196-9781(00)00221-7 10959010

[phy270561-bib-0026] Rosano, G. M. , Gebara, O. , Sheiban, I. , Silvestri, A. , Wajngarten, M. , Vitale, C. , Aldrighi, J. M. , Ramires, A. F. , Fini, M. , & Mercuro, G. (2007). Acute administration of 17β‐estradiol reduces endothelin‐1 release during pacing‐induced ischemia. International Journal of Cardiology, 116(1), 34–39. 10.1016/j.ijcard.2006.03.025 16814412

[phy270561-bib-0027] Sarafidis, P. A. , & Bakris, G. L. (2007). Review: Insulin and endothelin: An interplay contributing to hypertension development? Journal of Clinical Endocrinology and Metabolism, 92(2), 379–385. 10.1210/jc.2006-1819 17118997

[phy270561-bib-0028] Shoemaker, L. N. , Haigh, K. M. , Kuczmarski, A. V. , McGinty, S. J. , Welti, L. M. , Hobson, J. C. , Edwards, D. G. , Feinberg, R. F. , & Wenner, M. M. (2021). ETB receptor‐mediated vasodilation is regulated by estradiol in young women. American Journal of Physiology—Heart and Circulatory Physiology, 321(3), H592–H598. 10.1152/ajpheart.00087.2021 34415188 PMC8461841

[phy270561-bib-0029] Singer, K. , Maley, N. , Mergian, T. , DelProposto, J. , Cho, K. W. , Zamarron, B. F. , Martinez‐Santibanez, G. , Geletka, L. , Muir, L. , Wachowiak, P. , Demirjian, C. , & Lumeng, C. N. (2015). Differences in hematopoietic stem cells contribute to sexually dimorphic inflammatory responses to high fat diet‐induced obesity. Journal of Biological Chemistry, 290(21), 13250–13262. 10.1074/jbc.M114.634568 25869128 PMC4505578

[phy270561-bib-0030] Stapleton, S. , Welch, G. , DiBerardo, L. , & Freeman, L. R. (2024). Sex differences in a mouse model of diet‐induced obesity: The role of the gut microbiome. Biology of Sex Differences, 15(1), 5. 10.1186/s13293-023-00580-1 38200579 PMC10782710

[phy270561-bib-0031] Stranahan, A. M. , Guo, D. H. , Yamamoto, M. , Hernandez, C. M. , Khodadadi, H. , Baban, B. , Zhi, W. , Lei, Y. , Lu, X. , Ding, K. , & Isales, C. M. (2023). Sex differences in adipose tissue distribution determine susceptibility to neuroinflammation in mice with dietary obesity. Diabetes, 72(2), 245–260. 10.2337/db22-0192 36367881 PMC9871229

[phy270561-bib-0032] Wang, C. Y. , & Liao, J. K. (2012). A mouse model of diet‐induced obesity and insulin resistance. Methods in Molecular Biology, 821, 421–433. 10.1007/978-1-61779-430-8_27 22125082 PMC3807094

[phy270561-bib-0033] Wen, X. , Zhang, B. , Wu, B. , Xiao, H. , Li, Z. , Li, R. , Xu, X. , & Li, T. (2022). Signaling pathways in obesity: Mechanisms and therapeutic interventions. Signal Transduction and Targeted Therapy, 7(1), 298. 10.1038/s41392-022-01149-x 36031641 PMC9420733

